# New Isocoumarin and Pyrone Derivatives from the Chinese Mangrove Plant *Rhizophora mangle*-Associated Fungus *Phomopsis* sp. DHS-11

**DOI:** 10.3390/molecules28093756

**Published:** 2023-04-27

**Authors:** Zhikai Guo, Biting Chen, Dandan Chen, Xiaoling Deng, Jingzhe Yuan, Shiqing Zhang, Zijun Xiong, Jing Xu

**Affiliations:** 1 Hainan Key Laboratory of Tropical Microbe Resources, Institute of Tropical Bioscience and Biotechnology, Chinese Academy of Tropical Agricultural Sciences & Key Laboratory for Biology and Genetic Resources of Tropical Crops of Hainan Province, Hainan Institute for Tropical Agricultural Resources, Haikou 571101, China; cbt688228@163.com (B.C.); yuanjingzhe@itbb.org.cn (J.Y.); zhangshiqing@itbb.org.cn (S.Z.); xiongzijun@itbb.org.cn (Z.X.); 2 School of Chemical Engineering and Technology, Hainan University, Haikou 570208, China; 17769570501@163.com (D.C.); 20081700210002@hainanu.edu.cn (X.D.)

**Keywords:** mangrove, endophytic fungus, *Phomopsis* sp., polyketides, cytotoxic activity

## Abstract

Mangrove-associated fungi are important sources for the discovery of new bioactive natural products. Three new isocoumarins (**1**–**3**) and one new pyrone derivative (**4**) were isolated from the ethyl acetate extract of the fermentation broth of the mangrove endophytic fungus *Phomopsis* sp. DHS-11. Nuclear magnetic resonance (NMR) spectroscopy (one-dimensional and two-dimensional) and mass spectrometry were used to determine the structures of these new compounds. The absolute configurations for the new isocoumarins **1**–**3** were determined by comparing their experimental and calculated electronic circular dichroism (ECD) spectra, while the configuration for the new pyrone-derivative **4** was tentatively solved by comparison of its ^13^C NMR data with reported data. In the biological activity test, compounds **1** and **3** showed cytotoxic activity against HeLa cells with IC_50_ values of 11.49 ± 1.64 µM and 8.70 ± 0.94 µM, respectively. The initial structure and activity relationship (SAR) analysis revealed that the length of the side chain at C-3 for isocoumarin-type compounds **1**–**3** could affect the cytotoxicity against HeLa cells. Compound **4** exhibited cytotoxic activities against human hepatoma cells HepG2 with an IC_50_ value of 34.10 ± 2.92 µM. All compounds have no immunosuppressive activity.

## 1. Introduction

Marine-derived fungi are important sources for the discovery of natural products with unique structures and significant pharmacological activities [1,2,3,4]. Mangrove plants grow in saline-alkali habitats at the junction of tropical and subtropical climates, land and ocean, and endophytic fungi inhabiting them are the second-largest ecological group among marine fungi [5,6,7,8]. Endophytes are a group of parasitic species living in the tissue of plants without causing any obvious pathogenic symptoms [9]. Mangrove endophytic fungi form prolific metabolic pathways and adaptive mechanisms in unique environments, which produces a large number of natural secondary metabolites with novel structures and biological significance, such as terpenes, polyketides, alkaloids, etc., attracting extensive attention in drug mining and agrochemical applications [10,11,12,13]. *Phomopsis* sp. is a ubiquitous fungus and research on the secondary metabolites of this genus from a chemical point of view has shown that a variety of biologically active products have been found [14,15], such as lung cancer prevention potential drug cytochalasin, antibacterial chromones, antifungal lactones, and cytotoxic sesquiterpenes, etc., [16,17,18,19,20].

**Figure 1:**
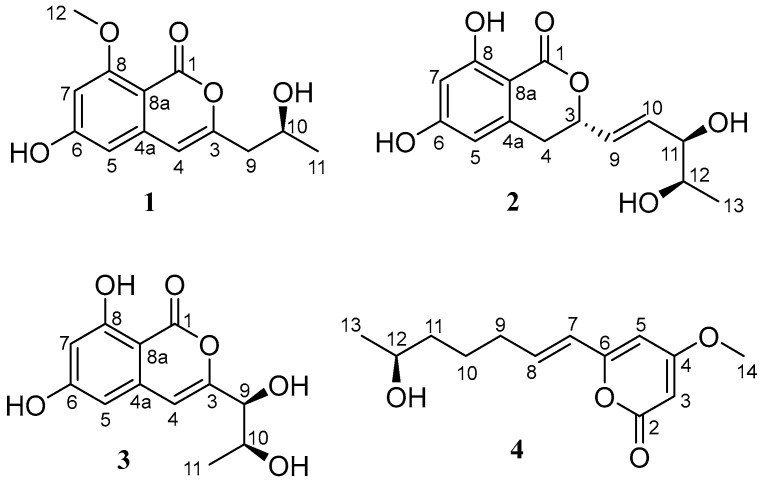
The chemical structures of compounds **1**–**4**.

Previously, our research group has found many bioactive secondary metabolites from mangrove-plant-derived endophytic fungi from the Dong Zhai Gang mangrove garden in Hainan [21,22,23]. Recently, the fungus *Phomopsis* sp. DHS-11 was isolated from the root of freshly harvested mangrove plant, *Rhizophora mangle*. Preliminary phytochemical investigation on the fermentation products of the mangrove endophyte *Phomopsis* sp. DHS-11 led to the discovery of norpestaphthalides and a cerebroside-type natural compound, alternaroside B; however, biological tests showed that none of them had cytotoxic or immunosuppressive activities [24]. As part of our ongoing exploration of new and bioactive natural product targets in endophytic fungi of Hainan mangrove plants, one new pyrone-type and three new isocoumarin-type polyketides were isolated from the fermentation broth of *Phomopsis* sp. DHS-11, including phomoisocoumarin E (**1**), phomodihydroisocoumarin A (**2**), phomoisocoumarin F (**3**), and phomopyrone D (**4**) (Figure 1). Herein, we report the isolation of these compounds, the elucidation of their structures, and their biological activities.

## 2. Results

### 2.1. Structure Elucidation of the New Compounds

**Figure 2:**
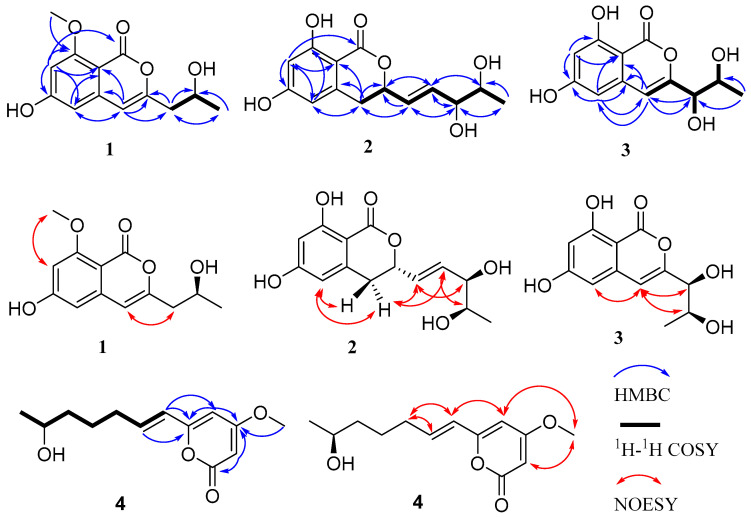
Key HMBC, ^1^H-^1^H COSY, and NOESY NMR correlations of compounds **1**−**4**.

**Figure 3:**
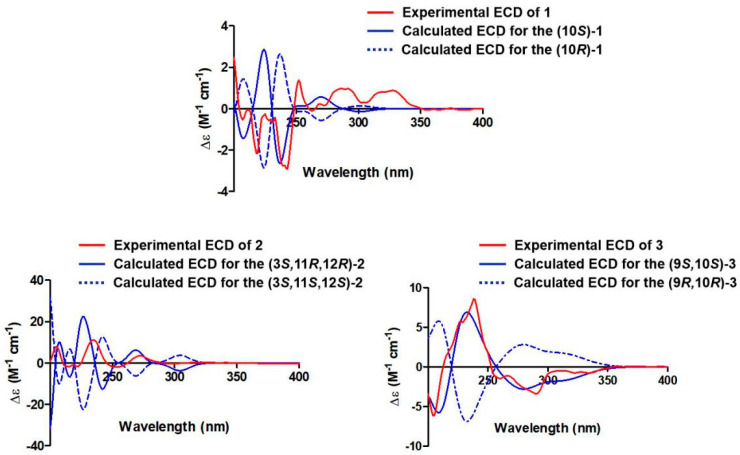
Comparison of experimental and calculated ECD spectra of compounds **1**–**3** in MeOH at the B3LYP/6-31+g (d, p) level.

Compound **1** was isolated as an amorphous powder. Its molecular formula of C_13_H_14_O_5_ was determined by high-resolution electrospray ionization mass spectroscopy (HRESIMS) [*m/z* 249.0774 [M − H]^−^ (calcd for C_13_H_13_O_5_, 249.0768], possessing seven degrees of unsaturation. According to the ^1^H NMR (Figure S1) data of compound **1** (Table 1), there were three aromatic proton signals at δ_H_ 6.25 (1H, s, H-4), 6.31 (1H, s, H-5), and 6.40 (1H, s, H-7). In addition, two methylene proton signals at δ_H_ 2.45 (1H, dd, *J* = 14.2, 7.1 Hz, H-9a) and 2.41 (1H, dd, *J* = 14.2, 5.6 Hz, H-9b), an oxygenated methine proton signal at 3.96 (1H, m, H-10), a methyl proton signal at δ_H_ 1.12 (3H, d, *J* = 6.2 Hz, H-11), a methoxyl proton signal at δ_H_ 3.80 (3H, s, H-12), and a hydroxyl group signal at δ_H_ 4.78 (1H, brs, OH-10) were present. The ^13^C NMR (Figure S2) and DEPT135 (Figure S3) data (Table 2) showed that compound **1** contained thirteen carbons, including an ester carbonyl carbon, eight aromatic or olefinic carbons (three of which are protonated), a methylene carbon, an oxygenated methine carbon, and two methyl carbons. The data for compound **1** were similar to those of pestalotiorin, which were reported in the literature [25]. The major difference was the absence of a methyl group at C-7 in compound **1**. The ^1^H-^1^H COSY correlations from H-10 to H_2_-9 and H_3_-11 and key HMBC correlations from H-4 to C-5, C-4a, C-8a, C-3, and C-9, from H-7 to C-1, C-5, C-6, C-8, and C-8a, and from H_3_-12 to C-8 were observed. Additionally, the methoxyl protons (H_3_-12) only showed NOE correlation with H-7, permitting the location of the methoxyl group at C-8. The overall analysis of the HSQC, HMBC, NOESY, and MS spectra (Figure 2 and Figures S4–S8) led to the full assignment of the structure as shown in Figure 1. We tried to use the modified Mosher’s method to determine the absolute configuration of C-10 in compound **1** but without success. Thus, the absolute configuration of C-10 was tentatively determined to be 10*S* based on its specific rotation and calculated ECD spectrum (Figure 3). Therefore, the structure of compound **1** was identified and named phomoisocoumarin E.

Compound **2** was obtained as a white oil. HRESIMS data at *m/z* 281.1012 [M + H]^+^ (calcd for C_14_H_17_O_6_, 281.1020) (Figure S16) showed the molecular formula of C_14_H_16_O_6_ with seven degrees of unsaturation. The IR absorption band at 3422 cm^−1^ suggested the presence of a hydroxyl group. The ^1^H NMR data (Table 1 and Figure S9) indicated the presence of two meta-coupled aromatic proton signals at δ_H_ 6.20 (1H, d, *J* = 1.8 Hz, H-7) and 6.27 (1H, d, *J* = 1.8 Hz, H-5), indicating the presence of a 1,2,3,5-tetrasubstituted aromatic ring. Analysis of the ^1^H, ^13^C (Table 2 and Figure S10), DEPT135 (Figure S11), and HSQC (Figure S12) NMR data revealed the presence of a methyl group, a methylene group, three oxygenated methine groups, eight aromatic or olefinic carbons (four of which are protonated), and an ester carbonyl group. Additionally, three hydroxyl proton signals at δ_H_ 11.09 (1H, s, 8-OH), 4.89 (1H, s, 11-OH or 12-OH), and 4.52 (1H, s, 11-OH or 12-OH) were observed. The ^1^H-^1^H COSY (Figure 2 and Figure S14) spectrum showed correlations from H-12 to H_3_-13 and H-11, from H-10 to H-9 and H-11, from H-9 to H-3 and H-10, and from H-3 to H-4 and H-9 (Figure 2). The HMBC spectra (Figure S13) correlations of H-9 with C-3 and C-4, H-7 with C-1, C-5, C-6, C-8, and C-8a, H-5 with C-4, C-7, and C-8a, and H_2_-4 with C-3, C-4a, C-5, C-8a, and C-9 revealed an isocoumarin ring system (Figure 2). Comprehensive NMR analysis of HSQC, HMBC, NOESY, and MS data allowed the assignment of structure as shown in Figure 2. The *E* configuration of the olefinic bond C9-C10 was deduced by the large coupling constant (*J*_H-9_/_H-10_ = 15.6 Hz). The absolute configuration at C-3, C-11, and C-12 was determined to be 3*S*, 11*R*, and 12*R* by comparing the calculated ECD spectrum with the experimental ECD spectrum (Figure 3). Therefore, the structure of compound **2** was determined and named phomodihydroisocoumarin A.

Compound **3** was isolated as a viscous oil. It has a molecular formula of C_12_H_12_O_6_ as determined by HRESIMS at *m/z* 275.0520 (calculated for C_12_H_12_NaO_6_, 275.0526 [M + Na]^+^) (Figure S24) and NMR spectrum (Figures S17–S23). Analysis of the ^1^H NMR data (Table 1) revealed one methyl group signal at δ_H_ 1.12 (1H, d, *J* = 6.2 Hz, H_3_-11), three aromatic proton signals at δ_H_ 6.59 (1H, s, H-4), 6.42 (1H, d, *J* = 1.9 Hz, H-5) and 6.33 (1H, d, *J* = 1.9 Hz, H-7), two oxygenated methine proton signals at δ_H_ 3.98 (1H, d, *J* = 6.5 Hz, H-9) and 3.80 (1H, qui, *J* = 6.3 Hz, H-10), and three hydroxyl proton signals at δ_H_ 11.00, 5.65, and 4.78. The ^13^C NMR combined with DEPT135 spectra (Table 2) showed a total of 12 carbon signals, including one methyl group, two oxygenated methine groups, eight aromatic or olefinic carbons (three of which are protonated), and one ester carbonyl group. The ^1^H and ^13^C NMR data of **3** were very similar to those of compound **1**. The distinction was attributed to the replacement of the methoxyl group at C-8 by a new phenolic hydroxyl group and the presence of a hydroxyl group at C-9 in **3**. The HMBC, ^1^H-^1^H COSY, and NOESY experiments (Figure 2) confirmed the deduction and allowed the assignment of structure as shown in Figure 1. Based on the analysis of the chemical shifts of C-9 (δ_C_ 74.8) and C-10 (δ_C_ 67.5) and the small coupling constant (*J*_H-11/H-12_ = 6.5 Hz), suggesting a *cis* configuration of C9 and C10 [26,27]. The relative configurations of C9 and C10 were also determined by NOESY correlations from H-4 to H-9 and H-10 (Figure 2). The absolute configurations of C9 and C10 were established as 9*S*,10*S* by comparison of their experimental ECD spectrum with the calculated ECD curves (Figure 3). Thus, compound **3** was identified as a new compound and named phomoisocoumarin F.

Compound **4** was obtained as an amorphous powder. Its molecular formula was deduced as C_13_H_18_O_4_ by its HRESIMS data at *m/z* 261.1095 [M + Na]^+^ (calcd for C_13_H_18_NaO_4_, 261.1097) (Figure S32), indicating five degrees of unsaturation. The ^1^H NMR spectrum (Figure S25) displayed one methyl proton signal at δ_H_ 1.06 (3H, d, *J* = 6.2 Hz, H-13), one methoxyl proton signal at δ_H_ 3.76 (3H, s, H-14), one oxygenated aliphatic methine proton signal at δ_H_ 3.64 (1H, m, H-12), four olefinic proton signals at δ_H_ 6.69 (1H, dt, *J* = 15.6, 7.2 Hz, H-8), 6.04 (1H, d, *J* = 15.6 Hz, H-7), 5.95 (1H, d, *J* = 2.1 Hz, H-5), 5.47 (1H, d, *J* = 2.1 Hz, H-3), and three sp^3^ methylene proton signals at δ_H_ 2.17 (2H, m, H-9), 1.66–1.48 (2H, m, H-10), 1.53–1.42 (2H, m, H-11) (Table 1). The ^13^C NMR spectrum (Figure S26) showed the presence of one methyl carbon signal at δ_C_ 23.5 (C-13) and one methoxyl carbon signal at δ_C_ 57.0 (C-14) (Table 2). The ^1^H-^1^H COSY spectrum (Figure S30) of **4** revealed a spin system of H-7/H-8/H_2_-9/H_2_-10/H_2_-11/H-12/H_3_-13 (Figure 2). The *E* configuration of H-7 and H-8 was deduced from the large coupling constant (*J*_H-7/H-8_ = 15.6 Hz). The typical ^13^C NMR data at δ_C_ 174.0 (C-4), 167.0 (C-2), 160.3 (C-6), 101.1 (C-5), 88.9 (C-3), and 57.0 (C-14) suggested the presence of a 6-substituted 4-methoxyl-2*H*-pyran-2-one (2-pyrone) moiety in **4** [28]. In the HMBC spectrum, key correlations from H-8 to C-6, from H-7 to C-5 and C-6, from H-5 to C-4 and C-6, and from H-3 to C-2 and C-4 allowed the connection of C-7 with C-6 on the 2-pyrone moiety (Figure 2). The methoxyl group positioned at C-4 of 2-pyrone moiety was secured by the observation of key HMBC correlations of H_3_-14 with C-4 and NOE correlations (Figure S31) of H_3_-14 with H-3 and H-5. The modified Mosher’s method was applied to determine the absolute configuration of C-12 in **4** but without success. The planar structure of **4** was very similar to scirpyrone D, possessing the same stereocenter with one hydroxyl group. Due to almost the same chemical shifts of the chiral carbons (δ_C_ 68.3 for C-12 in **4** and δ_C_ 68.1 for C-4′ in scirpyrone D) and opposite optical rotation values [−11.0 (*c* 0.10, MeOH) for **4** and 2.8 (*c* 0.60, MeOH) for scirpyrone D] [28], the absolute configuration of C-12 in **4** was determined to be 12*S*. Thus, the structure of **4** was elucidated as shown in Figure 1 and named phomopyrone D.

### 2.2. Biological Evaluation

The immunosuppressive and cytotoxic activities of compounds **1**–**4** were screened. For the cytotoxic activity test, all of these compounds (**1**–**4**) were first evaluated at the concentrations of 10 µg/mL. The result showed that only compounds **1**, **3,** and **4** had activity at this concentration. So the cytotoxic activities of these three compounds (**1**, **3,** and **4**) were evaluated in depth. The experimental results indicated that compounds **1** and **3** showed moderate inhibitory activities against human cervical cancer cells HeLa with IC_50_ values of 11.49 ± 1.64 µM and 8.70 ± 0.94 µM, respectively, which is less active than the positive drug doxorubicin with IC_50_ values of 0.95 ± 0.61 µM. The initial SAR analysis revealed that the length of the side chain at C-3 for isocoumarin-type compounds **1**–**3** could affect their cytotoxicity against HeLa cells, while the methoxyl modification at C-8 on the isocoumarin ring and the hydroxylation at C-9 on the side chain did not affect the activity. Simultaneously, only the pyrone derivative **4** exhibited significant cytotoxic activities against human hepatoma cells HepG2 with an IC_50_ value of 34.10 ± 2.92 µM, comparable with the positive drug 5-fluorouracil with an IC_50_ value of 21.69 ± 9.11 µM (Table 3), while all isolated isocoumarins **1**–**3** did not show activity when tested at the concentration of 10 µg/mL. However, all compounds have no immunosuppressive activity at the concentration of 10 µg/mL.

## 3. Materials and Methods

### 3.1. General Experimental Procedures

One-dimensional (1D) NMR (500 MHz for ^1^H NMR and 125 MHz for ^13^C NMR) and two-dimensional (2D) NMR (HSQC, HMBC, ^1^H-^1^HCOSY, ROSEY, or NOSEY) were measured on the Bruker AV-500 spectrometers (Bruker, Germany). The chemical shifts of ^1^H and ^13^C NMR data were given in δ (ppm) and referenced to the solvent signal (CD_3_OD, δ_H_ 3.31 and δ_C_ 49.00; DMSO-*d*_6_, δ_H_ 2.50 and δ_C_ 39.52). High-resolution electrospray ionization mass spectroscopy (HRESIMS) data were acquired on an Agilent 6210 time-of-flight LC-MS instrument (Agilent Technologies Inc., Palo Alto, CA, USA). Optical rotation values were measured by JASCO P-1020 digital polarimeter (JASCO, Tokyo, Japan). IR spectrum data were recorded on Nicolet 380 Infrared Spectrometer (Thermo Fisher, Waltham, MA, USA). The electronic circular dichroism (ECD) data were determined using JASCO J-715 Spectropolarimeter (Jasco, Tokyo, Japan). The semipreparative high-performance liquid chromatography (HPLC) was equipped with an ODS column (250.0 mm×10.0 mm, 5 μm, Thermo Fisher Scientific, Waltham, MA, USA). Column chromatography (CC) was performed on silica gel (60–80 mesh or 200–300 mesh; Qingdao Marine Chemical Inc., Qingdao, China), Sephadex LH-20 (PharmaciaBiotec, Uppsala, Sweden), and ODS (40–70 µm, Nacalai Tesque, Kyoto, Japan).

### 3.2. Fungal Material and Culture Conditions

The endophytic fungus strain *Phomopsis* sp. DHS-11 was isolated from the living root of the mangrove plant *Rhizophora mangle* collected in Dong Zhai Gang mangrove garden on Hainan Island, China, in October 2015. It was identified as *Phomopsis* sp. by ITS gene sequence (GenBank Accession no. MT126606) analysis [29]. This strain was deposited and maintained in the research group of one of the authors, J.X. The strain *Phomopsis* sp. DHS-11 was cultivated on PDA medium (potato extract 200 g/L, glucose 20 g/L, agar 15 g/L, chloramphenicol 0.1 g/L) at 28 °C for 6 days. Then the agar blocks with mycelia were added into 130 × 1000 mL Erlenmeyer flasks containing 100 g rice and 100 mL of seawater (1000 mL conical flask with 100 mL seawater), then fermented for 35 days.

### 3.3. Extraction and Isolation

After fermentation, the whole fermentation mixtures of *Phomopsis* sp. DHS-11 were collected and mashed with a glass rod and extracted three times with ethyl acetate at room temperature. Then, the whole organic solvent was concentrated in vacuo to obtain 80 g of crude extract. The crude extract was fully mixed and ground with silica gel (60–80 mesh), then subjected to silica gel (200–300 mesh) CC eluted by gradient elution of CH_2_Cl_2_/MeOH mixtures (*v/v*, 100:0, 100:1, 100:2, 100:4, 100:8, 100:16, 100:32, 100:64, 0:100) to obtain 9 fractions (Fr. 1–Fr. 9). The fraction Fr. 5 was eluted with gradient elution of CH_2_Cl_2_/MeOH mixtures (*v/v*, 100:0–100:32) to obtain 5 subfractions (Fr. 5.1–Fr. 5.5). The subfraction Fr. 5.2 was applied to ODS CC with gradient elution of MeOH/H_2_O mixtures (*v/v*, 1:4, 3:7, 2:3, 1:1, 3:2, 7:3, 4:1, 0:1) and obtained five subfractions (Fr. 5.2.1–Fr. 5.2.5). The subfraction Fr. 5.2.2 was conducted on HPLC (MeOH/H_2_O, 80:20, *v/v*; 3 mL/min, UV *λ*_max_ 254 nm) to obtain compound **1** (6 mg). The subfraction Fr. 5.3 was subjected to ODS CC eluted with gradient elution of MeOH/H_2_O (*v/v*, 1:4, 3:7, 2:3, 1:1, 3:2, 7:3, 4:1, 0:1) to yield subfractions Fr. 5.3.1–Fr. 5.3.4. The subfractions Fr. 5.3.1 and Fr. 5. 3.2 were, respectively, purified by HPLC (MeOH/H_2_O, 70:30 and 60:40, *v/v*; 3 mL/min, UV *λ*_max_ 254 nm) to yield compound **2** (4 mg) and compound **3** (5 mg); The fraction Fr. 6 was separated by silica gel CC eluted with gradient elution of CH_2_Cl_2_/MeOH mixtures (*v/v*, 100:0–100:16) to obtain three components (Fr. 6.1–Fr. 6.3). The subfraction Fr. 6.1 was subjected to ODS CC using gradient elution of MeOH/H_2_O (*v/v*, 1:4, 3:7, 2:3, 1:1, 3:2, 7:3, 4:1, 0:1) to give fractions. The subfraction Fr. 6.2 was subjected to ODS CC using gradient elution of MeOH/H_2_O to yield four subfractions (Fr. 6.2.1–Fr. 6.2.4). The subfraction Fr. 6.2.3 was purified by Sephadex LH-20 CC to yield two subfractions (Fr. 6.2.3.1 and Fr. 6.2.3.2). The subfraction Fr. 6.2.3.2 was further separated by semi-preparative HPLC (MeOH/H_2_O, 35:65, *v/v*; 3 mL/min, UV *λ*_max_ 254 nm) to afford compound **4** (2 mg). 

Compound **1**: amorphous powder, ${\left[\alpha \right]}_{D}^{25}$ + 3.000 (*c* 0.10, MeOH); CD (*c* 0.05, MeOH) *λ*_max_ (∆*ε*): 209 (−0.10), 211 (+0.04), 218 (−0.42), 237 (−0.49), 239 (−0.36), 243 (+0.34), 252 (+0.23) nm; IR (KBr) *υ*_max_: 3424, 2923, 1700, 1654, 1600, 1441, 1373, 1160 cm^−1^; ^1^H and ^13^C NMR data, see Table 1 and Table 2; HRESIMS *m/z* 249.0774 [M − H]^−^ (calcd for C_13_H_13_O_5_ 249.0768).

Compound **2**: white oil, ${\left[\alpha \right]}_{D}^{25}$ + 7.000 (*c* 0.10, MeOH); CD (*c* 0.05, MeOH) *λ*_max_ (∆*ε*): 206 (+1.35), 234 (+1.92), 255 (−0.34), 272 (+0.60), 300 (−0.05) nm; IR (KBr) *υ*_max_: 3422, 2925, 1630, 1517, 1466, 1381, 1167 cm^−1^; ^1^H and ^13^C NMR data, see Table 1 and Table 2; HRESIMS *m/z* 281.1012 [M + H]^+^ (calcd for C_14_H_17_O_6_, 281.1020).

Compound **3**: viscous oil, ${\left[\alpha \right]}_{D}^{25}$ − 20.000 (*c* 0.10, MeOH); CD (*c* 0.05, MeOH) *λ*_max_ (∆*ε*): 209 (−2.98), 237 (+6.50), 322 (−0.56) nm; IR (KBr) *υ*_max_: 3422, 2923, 1681, 1628, 1460, 1382, 1238, 1170 cm^−1^; ^1^H and ^13^C NMR data, see Table 1 and Table 2; HRESIMS *m/z* 275.0520 [M + Na]^+^ (calcd for C_12_H_12_NaO_6_, 275.0526).

Compound **4**: amorphous powder, ${\left[\alpha \right]}_{D}^{25}$ − 11.000 (*c* 0.10, MeOH); CD (*c* 0.05, MeOH) *λ*_max_ (∆*ε*): 205 (−0.16), 211 (−0.75), 218 (+0.11), 226 (−1.37) nm; IR (KBr) *υ*_max_: 3450, 2923, 1689, 1653, 1616, 1454, 1410, 1159 cm^−1^; ^1^H and ^13^C NMR data, see Table 1 and Table 2; HRESIMS *m/z* 261.1095 [M + Na]^+^ (calcd for C_13_H_18_NaO_4_, 261.1097).

### 3.4. Electronic Circular Dichroism (ECD) Calculation Details

Monte Carlo conformational searches were conducted by means of Spartan’s 14 software using Merck Molecular Force Field (MMFF). The conformers with Boltzmann-population of over 5% were chosen for ECD calculations, and then the conformers were initially optimized at B3LYP/6-31g level in gas. The theoretical calculations of ECD were carried out in MeOH using time-dependent density functional theory (TD-DFT) at the B3LYP/6-31+g (d, p) level for all conformers of compounds **1–3**. Rotatory strengths for a total of 30 excited states were calculated. ECD spectra were generated using the program SpecDis 1.6 (University of Würzburg, Würzburg, Germany) and GraphPad Prism 5 (University of California San Diego, San Diego, CA, USA) from dipole-length rotational strengths by applying Gaussian band shapes with sigma = 0.3 eV.

### 3.5. Cell Viability Assay

The antitumor activity of all isolated compounds **1**–**4** was evaluated as previously reported by using the MTT assay method [30]. At first, the antitumor activity of all of these isolated compounds (**1**–**4**) was evaluated at the concentrations of 10 µg/mL. Doxorubicin and 5-fluorouracil were used as positive control for human cervical cancer cells HeLa and human hepatoma cells HepG2, respectively. The prepared concentrations for each of compounds **1**, **3**, **4**, and positive drugs in tests were 10, 5, 2.5, 1, 0.5, 0.1, 0.05, and 0.01 µg/mL, while compound **2** was only evaluated at the concentration of 10 µg/mL as it displayed no activity. All cell lines were obtained from the Shanghai Cell Bank of the Chinese Academy of Sciences. The IC_50_ values of these compounds and positive controls were calculated after 24 h for HeLa cells and 48 h for HepG2 cells. 

### 3.6. Immunosuppressive Assay

The immunosuppressive activity testing of compounds **1**–**4** was conducted with the previously reported CCK-8 assay method [30]. In the test, we set up three parallel trials, using cyclosporin A as the positive control, experiments of concanavalin A (ConA)-induced T cells, and lipopolysaccharide (LPS)-induced B cells. The concentrations of compounds **1**–**4**, and cyclosporin A, ConA, and LPS were 10, 5, and 10 µg/mL, respectively. All mice were donated by the Hainan Medical College.

## 4. Conclusions

To summarize, four previously undescribed polyketides, including three new isocoumarins (**1**–**3**) and one new pyrone derivative (**4**), were obtained from the mangrove-derived fungus *Phomopsis* sp. DHS-11. The structures of these isolated compounds were determined by analysis of HRESIMS, 1D- and 2D-NMR, and ECD data. The antitumor activity assay suggested that the new pyrone compound **4** exhibited cytotoxic activities against human hepatoma cells HepG2 with an IC_50_ value of 34.10 ± 2.92 µM, comparable with the positive drug 5-fluorouracil, indicating that this new compound has the potential to develop novel anti-hepatoma drugs and deserves further study. In addition, two new isocoumarin-type compounds **1** and **3** showed inhibitory activities against human cervical cancer cells HeLa with IC_50_ values of 11.49 ± 1.64 µM and 8.70 ± 0.94 µM, respectively. In general, the results of this study expand the diversity of chemical constituents and biological activities isolated from the secondary metabolites of *Phomopsis* sp. DHS-11, and may provide new potential molecules for antitumor drug discovery. These results also prove that mangrove-associated fungi are still pools for mining new bioactive natural molecules.

## Figures and Tables

**Table 1:** ^1^H NMR (500 MHz) spectroscopic data of compounds **1**–**4**.

Position	1 ^a^	2 ^a^	3 ^a^	4 ^b^
	δ_H_, Mult. (*J* in Hz)	δ_H_, Mult. (*J* in Hz)	δ_H_, Mult. (*J* in Hz)	δ_H_, Mult. (*J* in Hz)
3		5.14, ddd (10.0, 5.6, 4.3)		5.47, d (2.1)
4	6.25,s	3.00, dd (12.7, 3.8); 2.92, dd (16.4, 10.1)	6.59, s	
5	6.31, s	6.27, d (1.8)	6.42, d (1.9)	5.95, d (2.1)
7	6.40, s	6.20, d (1.8)	6.33, d (1.9)	6.04, d (15.6)
8				6.69, dt (15.6, 7.2)
9	2.45, dd (14.2, 7.1); 2.41, dd (14.2, 5.6)	5.78, dd (15.6, 6.5)	3.98, d (6.5)	2.17, m
10	3.96, m	5.98, dd (15.6, 5.0)	3.80, qui (6.3)	1.66–1.58, m; 1.57–1.48, m
11	1.12, d (6.2)	3.79, t (4.9)	1.12, d (6.2)	1.53–1.42, m
12	3.80, s	3.45, qui (6.0)		3.64, m
13		1.01, d (6.3)		1.06, d (6.2)
14				3.76, s
8-OH		11.09, br s	11.00, s	
9-OH			5.65, s	
10-OH	4.78, br s		4.78, s	
11-OH		4.89, br s		
12-OH		4.52, br s		

^a^ Recorded in DMSO-*d*_6_; ^b^ Recorded in CD_3_OD.

**Table 2:** ^13^C NMR (125 MHz) spectroscopic data of compounds **1**–**4**.

Position	1 ^a^	2 ^a^	3 ^a^	4 ^b^
	δ_C_, Type	δ_C_, Type	δ_C_, Type	δ_C_, Type
1	165.0, C	169.1, C	166.0, C	
2				167.0, C
3	155.7, C	78.4, CH	157.6, C	88.9, CH
4	104.1, CH	32.5,CH_2_	104.6, CH	174.0, C
4a	141.7, C	141.7, C	139.3, C	
5	102.8, CH	107.1, CH	103.2, CH	101.1, CH
6	158.0, C	164.7, C	162.6, C	160.3, C
7	98.9, CH	100.9, CH	101.7, CH	122.8, CH
8	163.1, C	163.4, C	165.4, C	140.9, CH
8a	100.3, C	100.1, C	98.3, C	
9	42.7,CH_2_	126.5, CH	74.8, CH	33.6, CH_2_
10	63.9,CH	135.5, CH	67.5, CH	25.9, CH_2_
11	23.4, CH_3_	74.5, CH	19.2, CH_3_	39.5, CH_2_
12	55.7, CH_3_	69.6, CH		68.3, CH
13		19.0, CH_3_		23.5, CH_3_
14				57.0, CH_3_

^a^ Recorded in DMSO-*d*_6_; ^b^ Recorded in CD_3_OD.

**Table 3:** Cytotoxic activities of compounds **1**, **3**, and **4** (IC_50_, µM).

Compounds	HeLa	HepG2
**1**	11.49 ± 1.64	--
**3**	8.70 ± 0.94	--
**4**	--	34.10 ± 2.92
Doxorubicin	0.95 ± 0.61	-
5-Fluorouracil	-	21.69 ± 9.11

“--” no activity at the concentration of 10 µg/mL; “-” no data.

## Data Availability

The authors declare that all relevant data supporting the results of this study are available within the article and its Supplementary Materials file, or from the corresponding authors upon request.

## Supplementary Materials

The following supporting information can be downloaded at: https://www.mdpi.com/article/10.3390/molecules28093756/s1, Figures S1–S32: 1D-, 2D-NMR, and MS spectra of compounds **1**–**4**.

## Sample Availability

Samples of compounds **1**–**4** are available from the authors.
