# Supplementary material for: New Isocoumarin and Pyrone Derivatives from the Chinese Mangrove Plant Rhizophora mangle-Associated Fungus Phomopsis sp. DHS-11
Source: Molecules. 2023 Apr 27;28(9):3756. doi: 10.3390/molecules28093756 (PMC10180500; doi:10.3390/molecules28093756)
Supplement: Supplementary file 1 [file molecules-28-03756-s001.zip › molecules-2359043-supplementary.pdf]

Figure S1. ^{1}H NMR (500 MHz, DMSO-d_6) spectrum of new compound **1**

Figure S2. ^{13}C NMR (125 MHz, DMSO-d_6) spectrum of new compound **1**

Figure S3. DEPT135 spectrum of new compound **1**

Figure S4. HSQC spectrum of new compound **1**

Figure S5. HMBC spectrum of new compound **1**

Figure S6. ^{1}H-^{1}H COSY spectrum of new compound **1**

Figure S7. NOESY spectrum of new compound **1**

Figure S8. The HRESIMS spectrum of the new compound **1**

Figure S9. ^{1}H NMR (500 MHz, DMSO-d_6) spectrum of new compound **2**

Figure S10. ^{13}C NMR (125 MHz, DMSO-d_6) spectrum of new compound **2**

Figure S11. DEPT135 spectrum of new compound **2**

Figure S12. HSQC spectrum of new compound **2**

Figure S13. HMBC spectrum of new compound **2**

Figure S14. ^{1}H-^{1}H COSY spectrum of new compound **2**

Figure S15. NOESY spectrum of new compound **2**

Figure S16. The HRESIMS spectrum of new compound **2**

Figure S17. ^{1}H NMR (500 MHz, DMSO-d_6) spectrum of new compound **3**

Figure S18. ^{13}C NMR (125 MHz, DMSO-d_6) spectrum of new compound **3**

Figure S19. DEPT135 spectrum of new compound **3**

Figure S20. HSQC spectrum of new compound **3**

Figure S21. HMBC spectrum of new compound **3**

Figure S22. ^{1}H-^{1}H COSY spectrum of new compound **3**

Figure S23. NOESY spectrum of new compound **3**

Figure S24. The HRESIMS spectrum of new compound **3**

Figure S25. ^{1}H NMR (500 MHz, CD_3OD) spectrum of new compound **4**

Figure S26. ^{13}C NMR (125 MHz, CD_3OD) spectrum of new compound **4**

Figure S27. DEPT135 spectrum of new compound **4**

Figure S28. HSQC spectrum of new compound **4**

Figure S29. HMBC spectrum of new compound **4**

Figure S30. ^{1}H-^{1}H COSY spectrum of new compound **4**

Figure S31. NOESY spectrum of new compound **4**

Figure S32. The HRESIMS spectrum of new compound **4**

Figure S1. ^{1}H NMR (500 MHz, DMSO-d_6) spectrum of new compound **1**

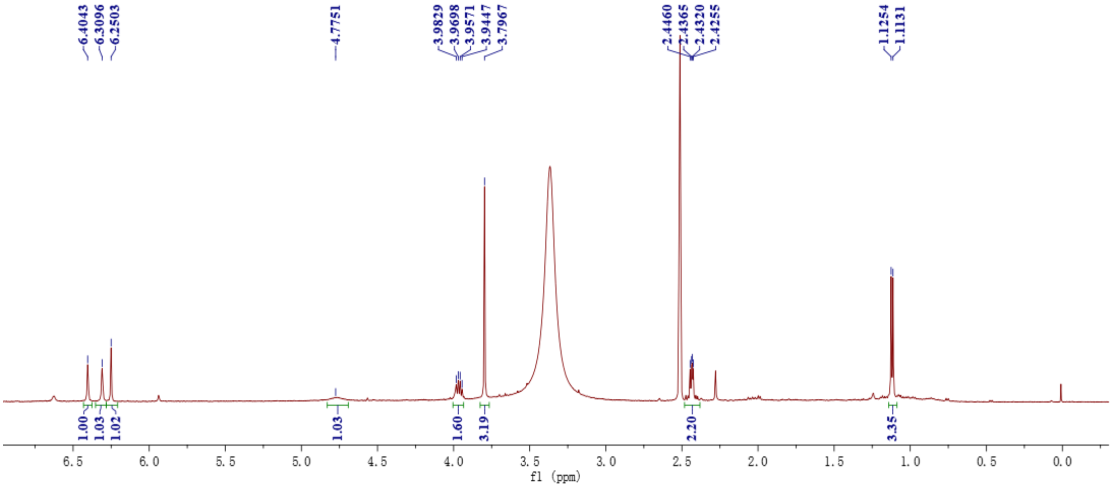

Figure S2. ^{13}C NMR (125 MHz, DMSO-d_6) spectrum of new compound **1**

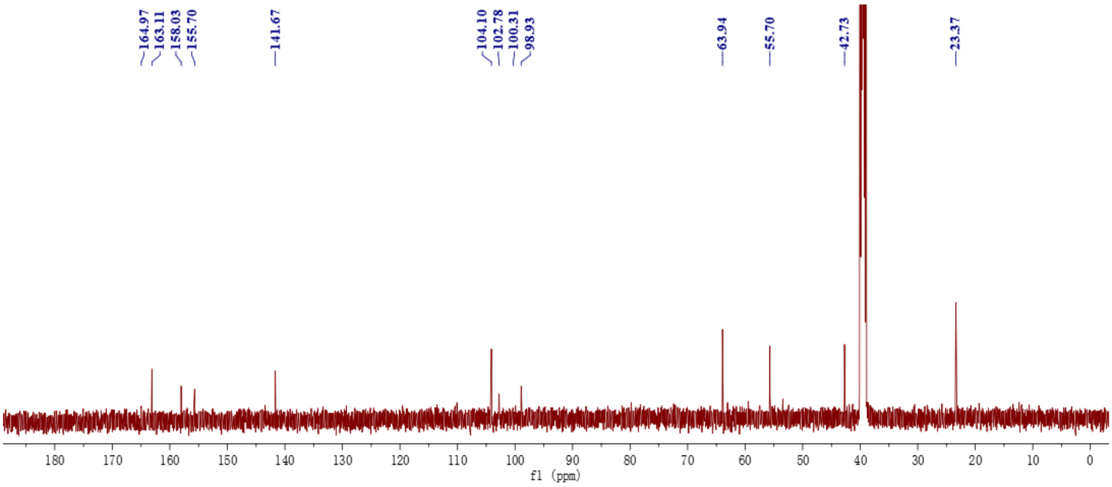

Figure S3. DEPT135 spectrum of new compound **1**

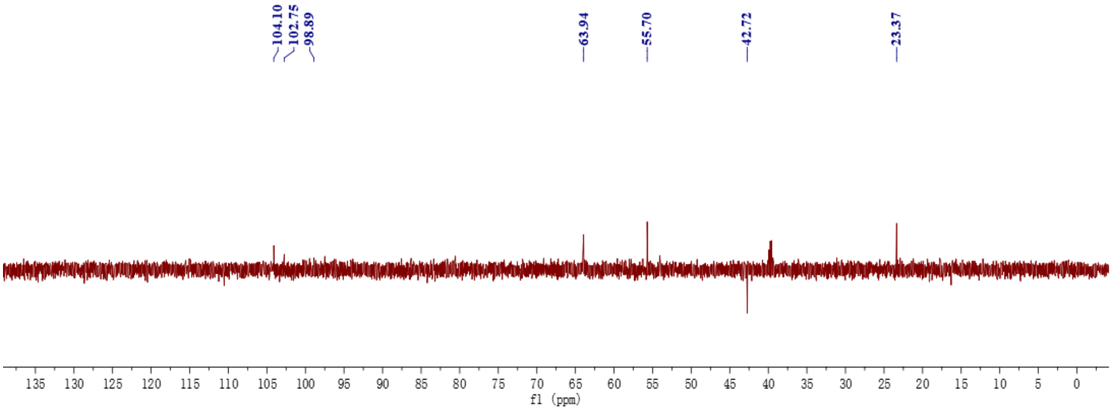

Figure S4. HSQC spectrum of new compound **1**

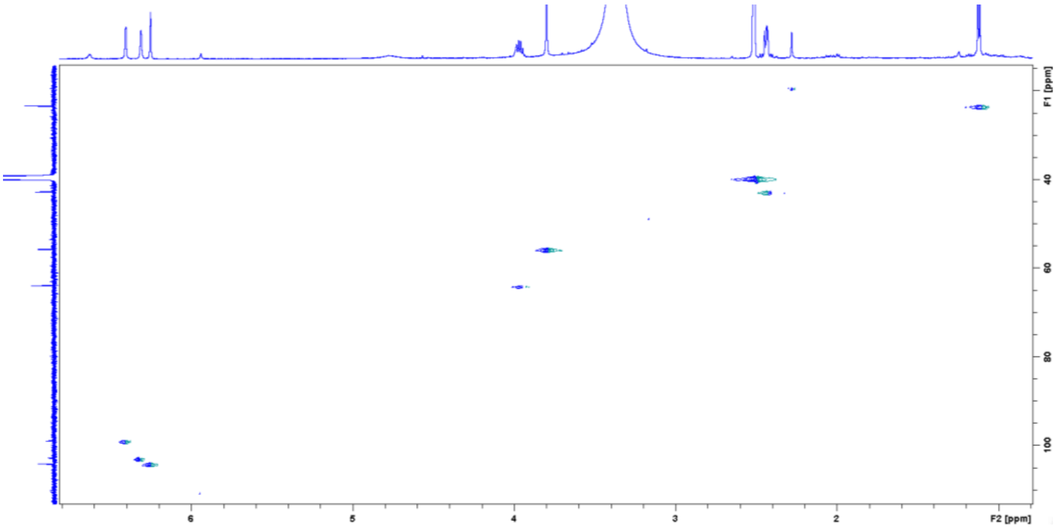

Figure S5. HMBC spectrum of new compound **1**

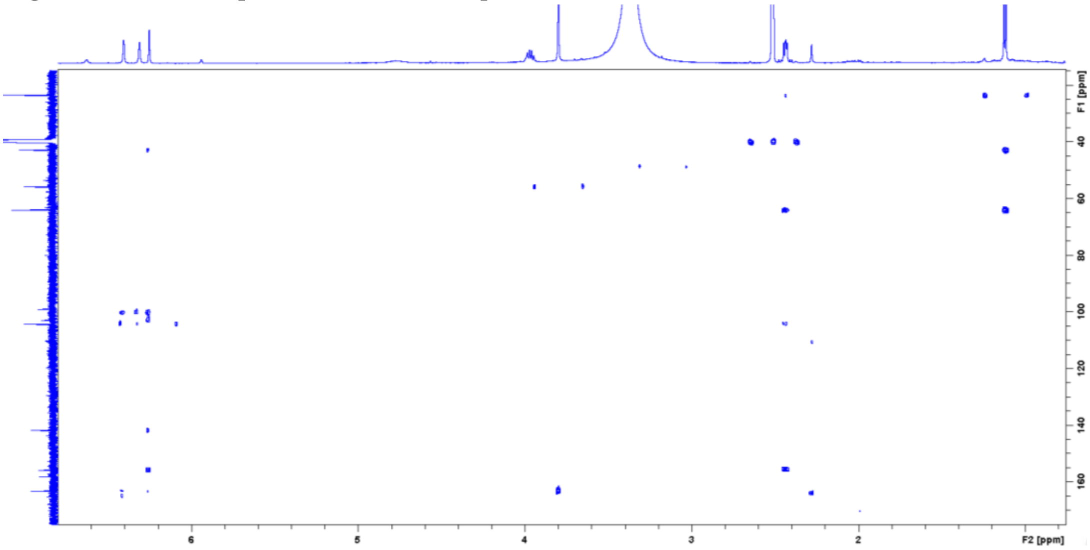

Figure S6. ^{1}H-^{1}H COSY spectrum of new compound **1**

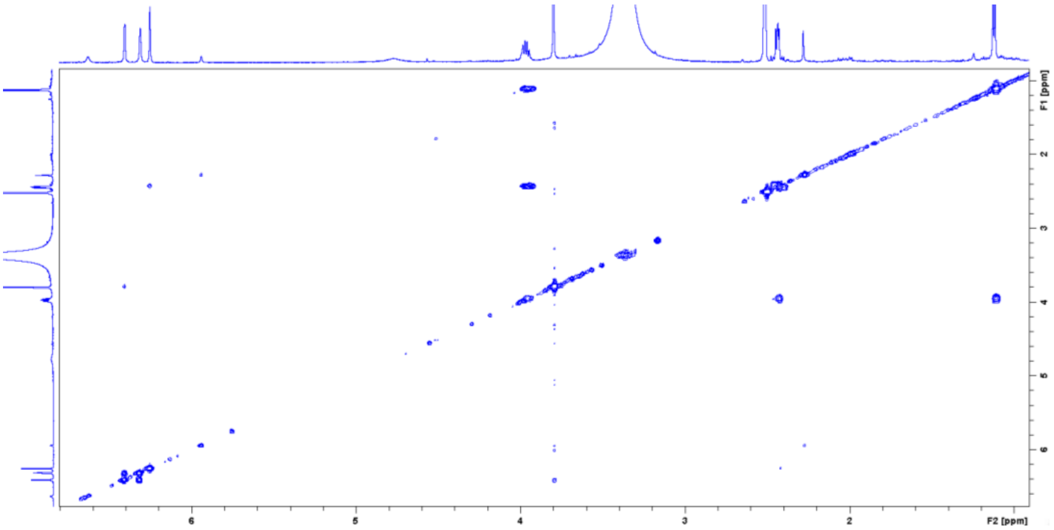

Figure S7. NOESY spectrum of new compound **1**

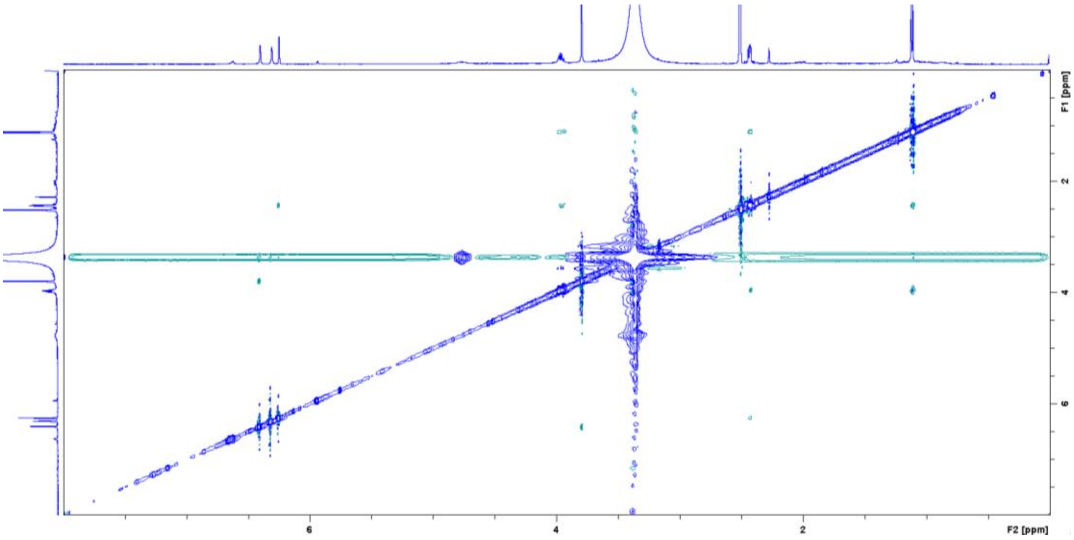

Figure S8. The HRESIMS spectrum of the new compound **1** (m/z 249.0774 $[M-H]^-$ (calcd for $C_{13}H_{13}O_5$ 249.0768))

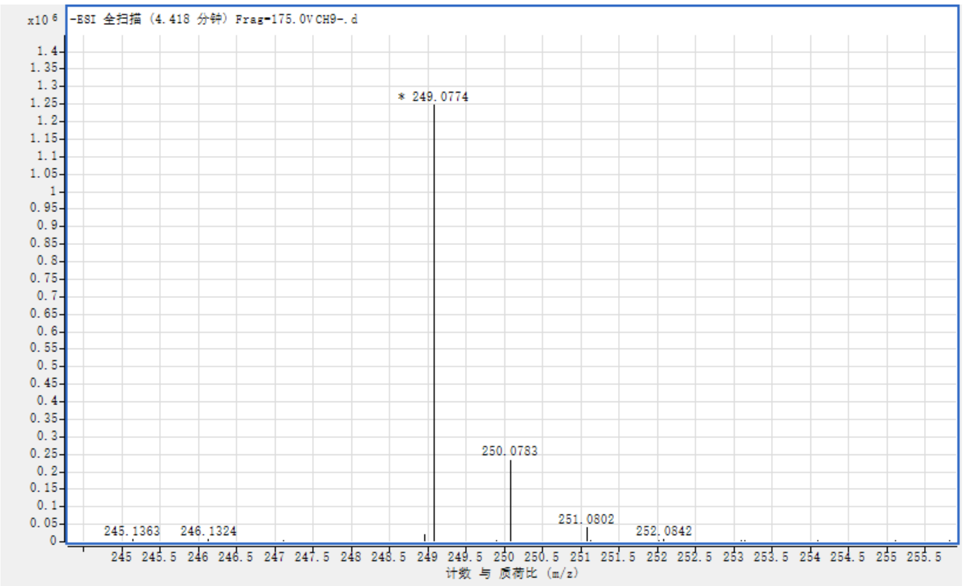

Figure S9. ^{1}H NMR (500 MHz, DMSO-d_6) spectrum of new compound **2**

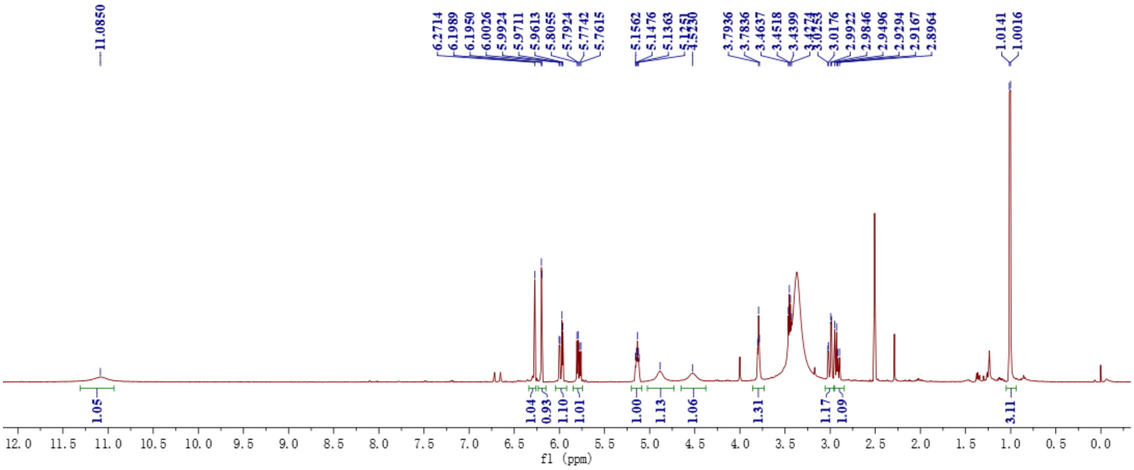

Figure S10. ^{13}C NMR (125 MHz, DMSO-d_6) spectrum of new compound **2**

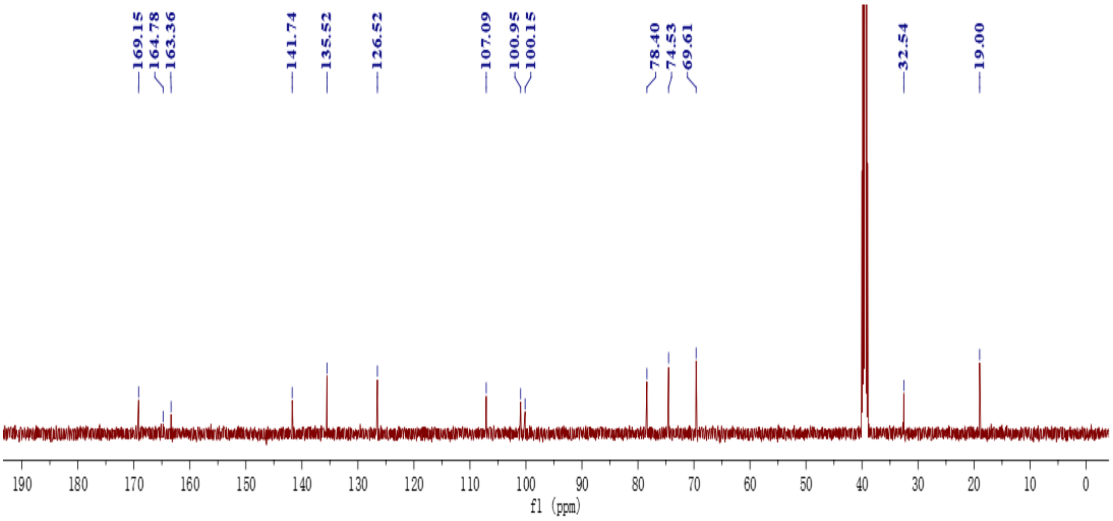

Figure S11. DEPT135 spectrum of new compound **2**

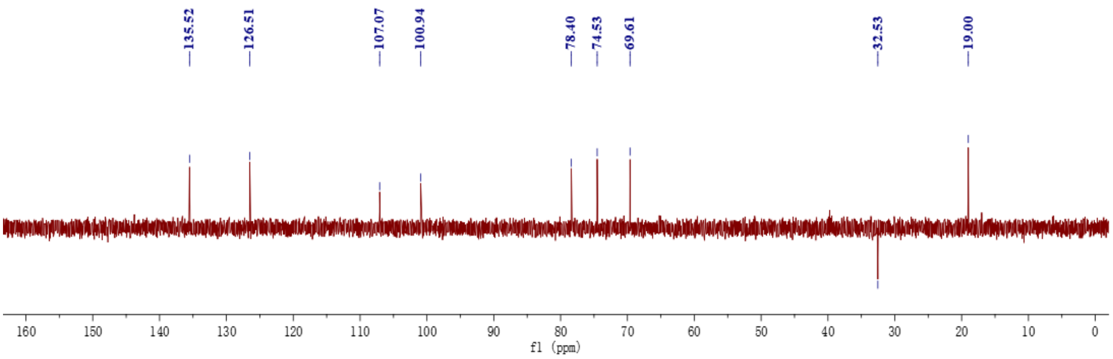

Figure S12. HSQC spectrum of new compound **2**

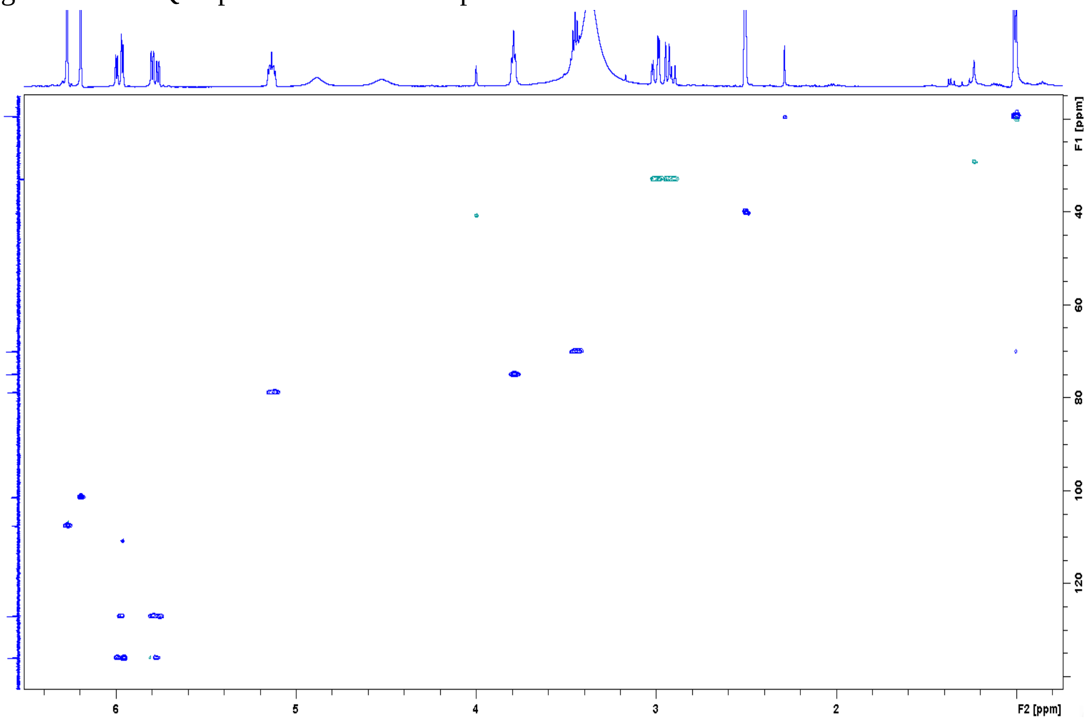

Figure S13. HMBC spectrum of new compound **2**

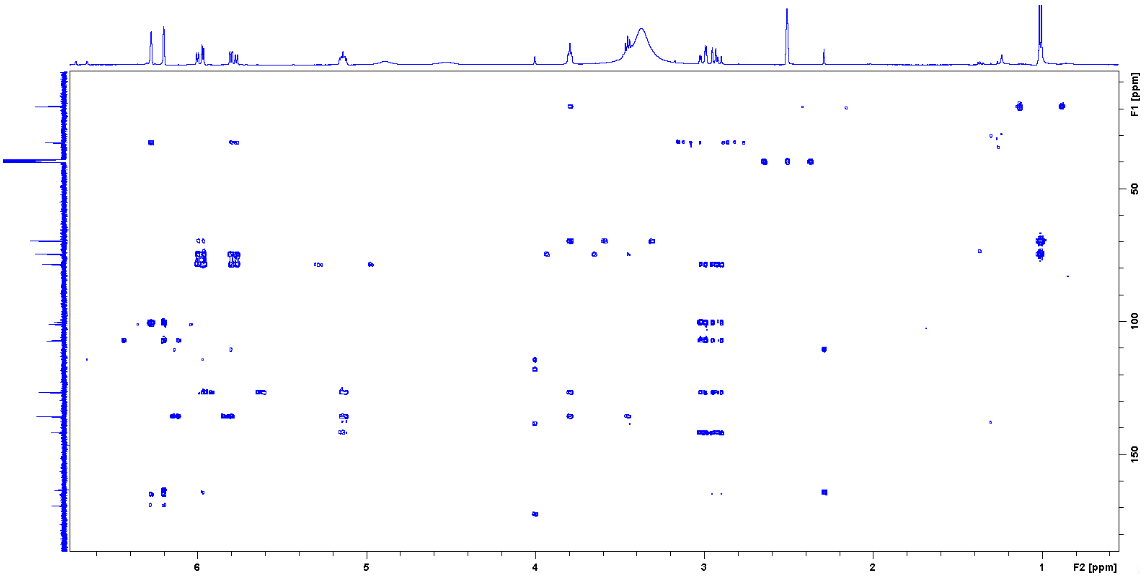

Figure S14. ^{1}H-^{1}H COSY spectrum of new compound **2**

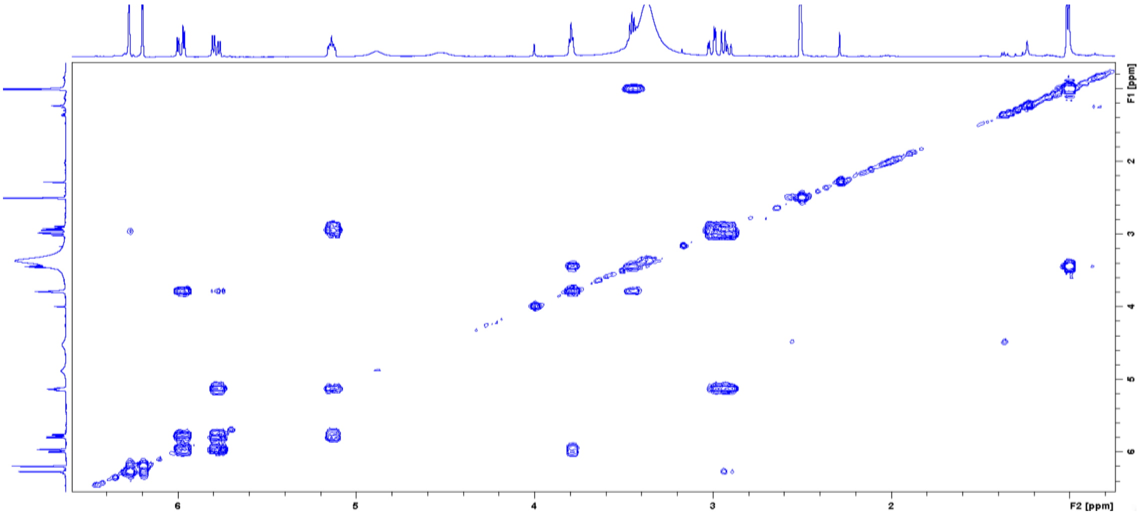

Figure S15. NOESY spectrum of new compound **2**

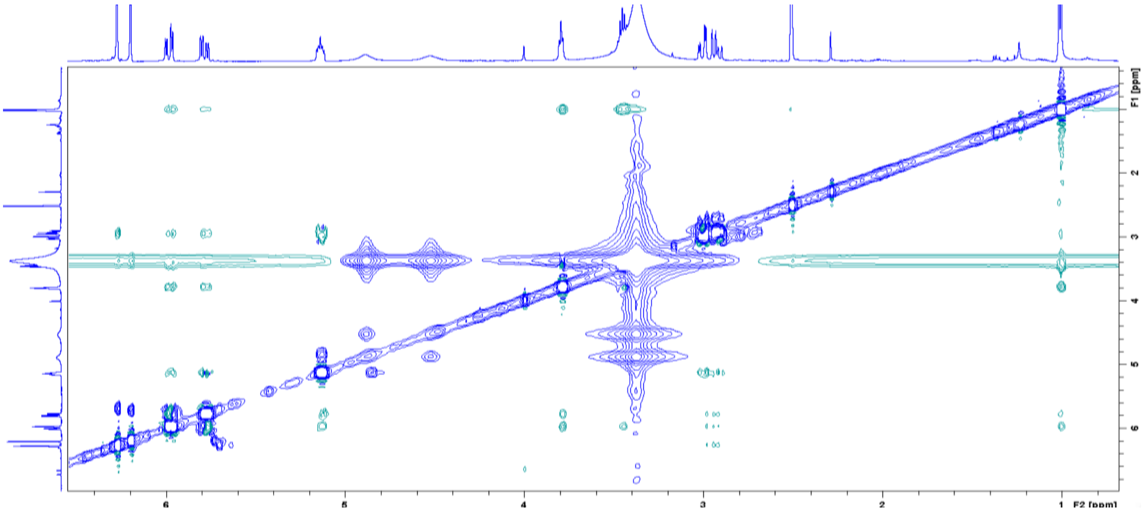

Figure S16. The HRESIMS spectrum of new compound **2** (*m/z* 281.1012 $[M+H]^+$ (calcd for $C_{14}H_{17}O_6$, 281.1020))

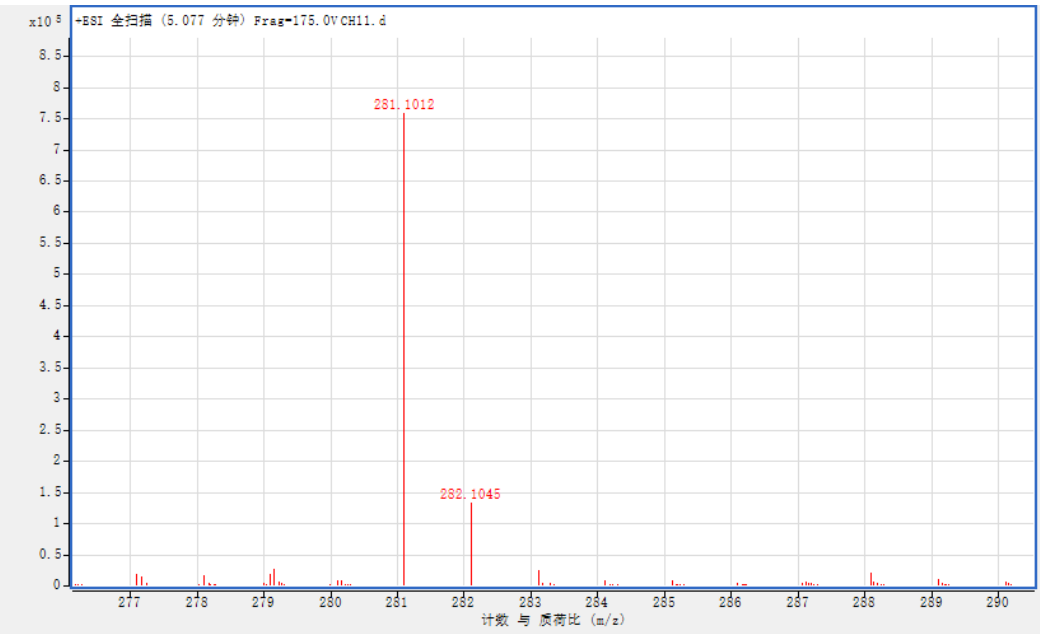

Figure S17. ^1H NMR (500 MHz, DMSO-d_6) spectrum of new compound **3**

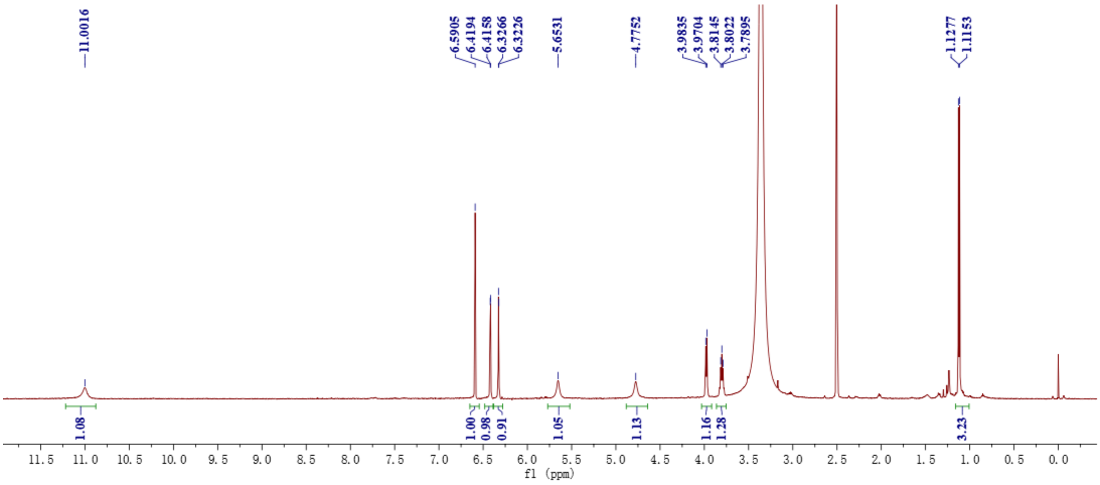

Figure S18. ^{13}C NMR (125 MHz, DMSO-d_6) spectrum of new compound **3**

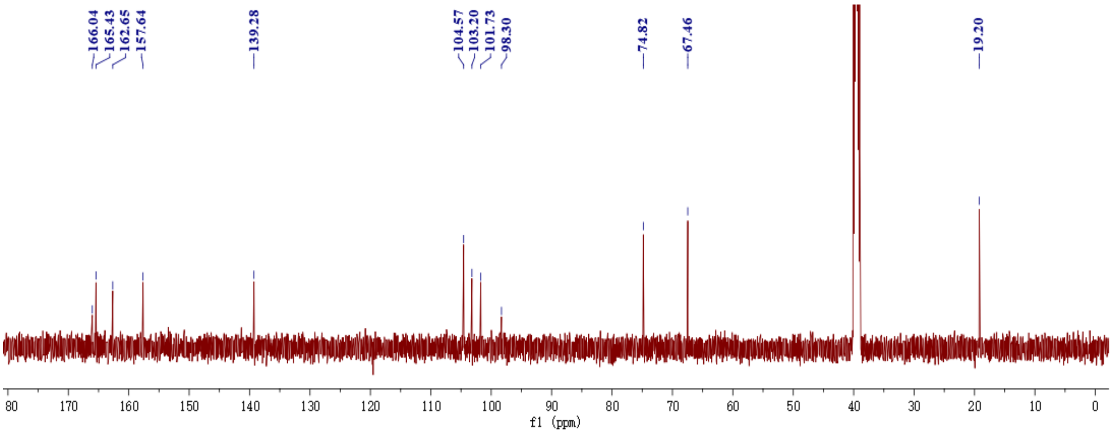

Figure S19. DEPT135 spectrum of new compound **3**

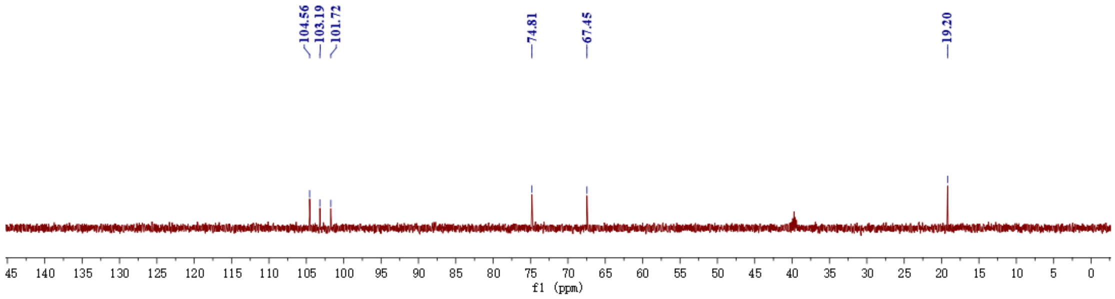

Figure S20. HSQC spectrum of new compound **3**

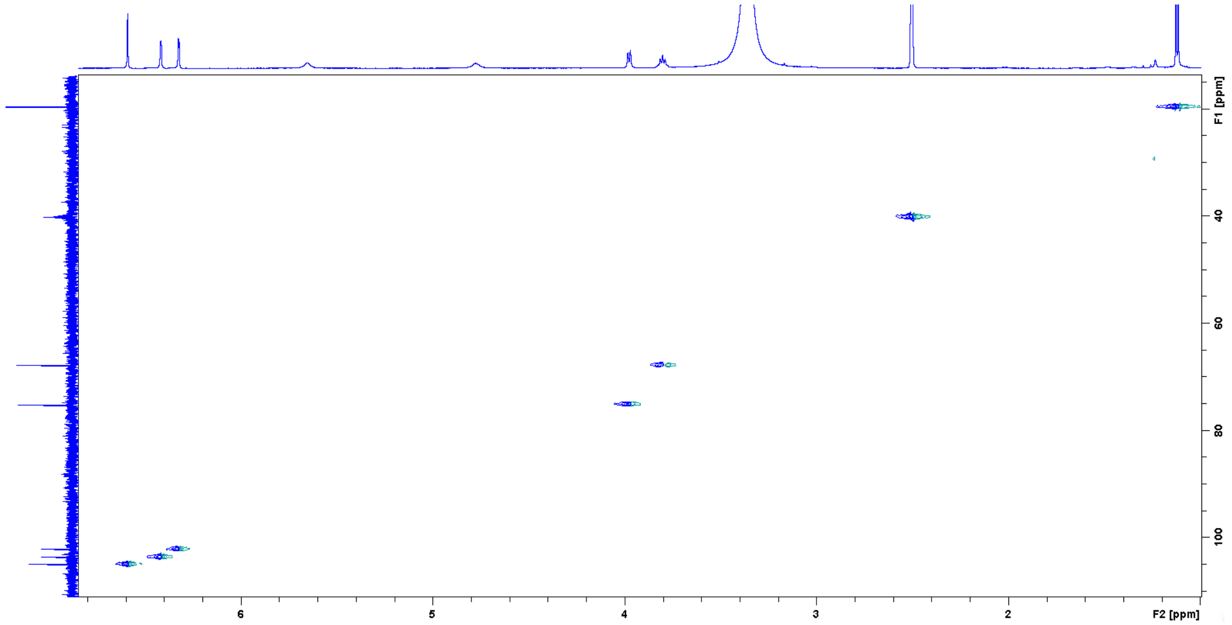

Figure S21. HMBC spectrum of new compound **3**

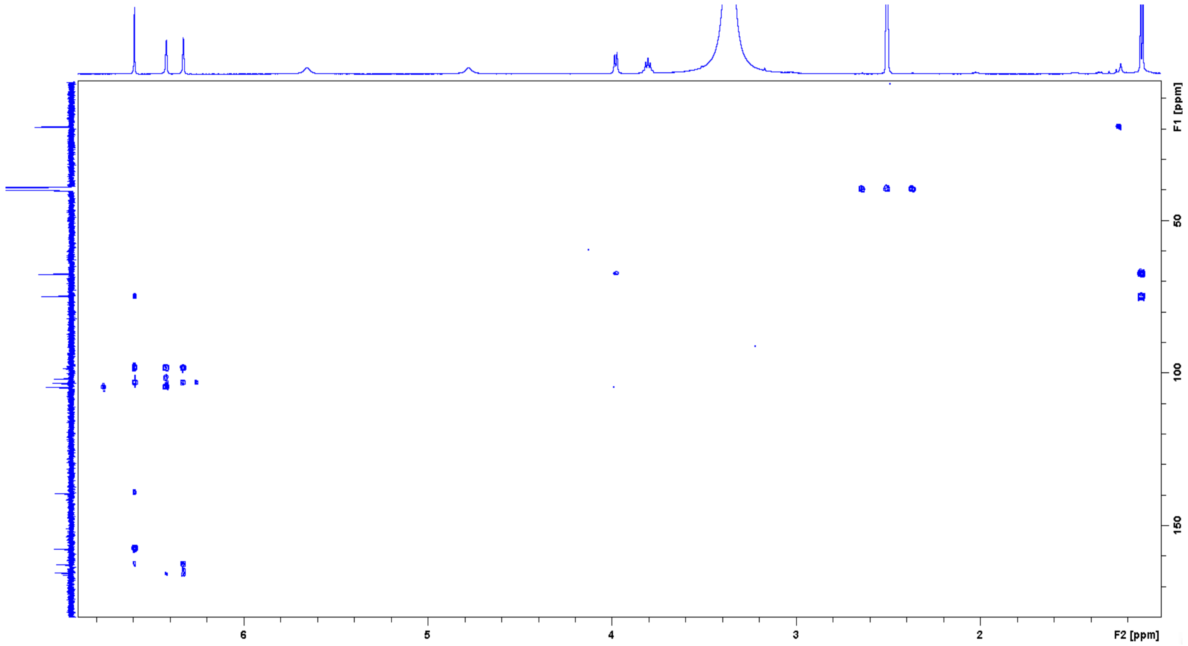

Figure S22. ^{1}H-^{1}H COSY spectrum of new compound **3**

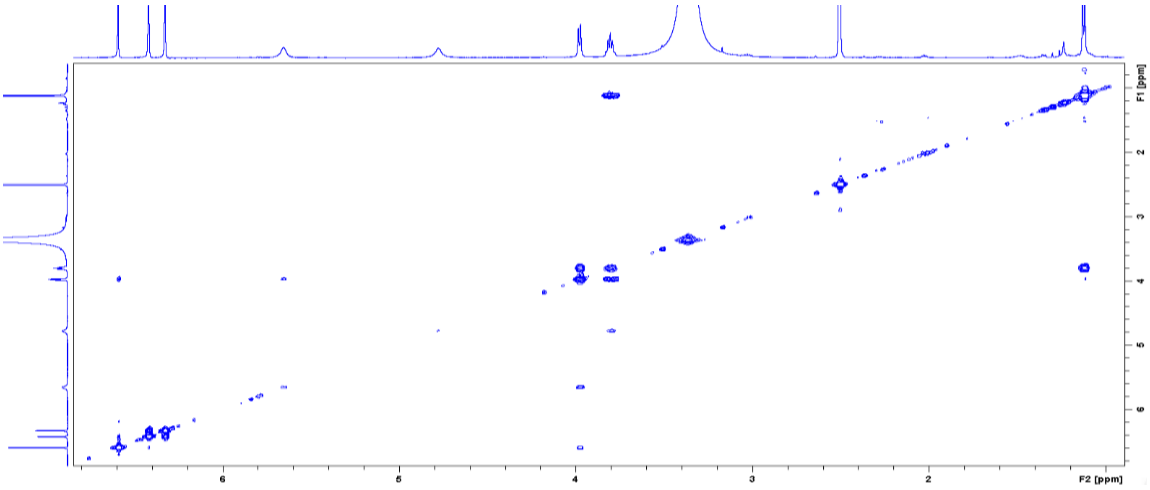

Figure S23. NOESY spectrum of new compound **3**

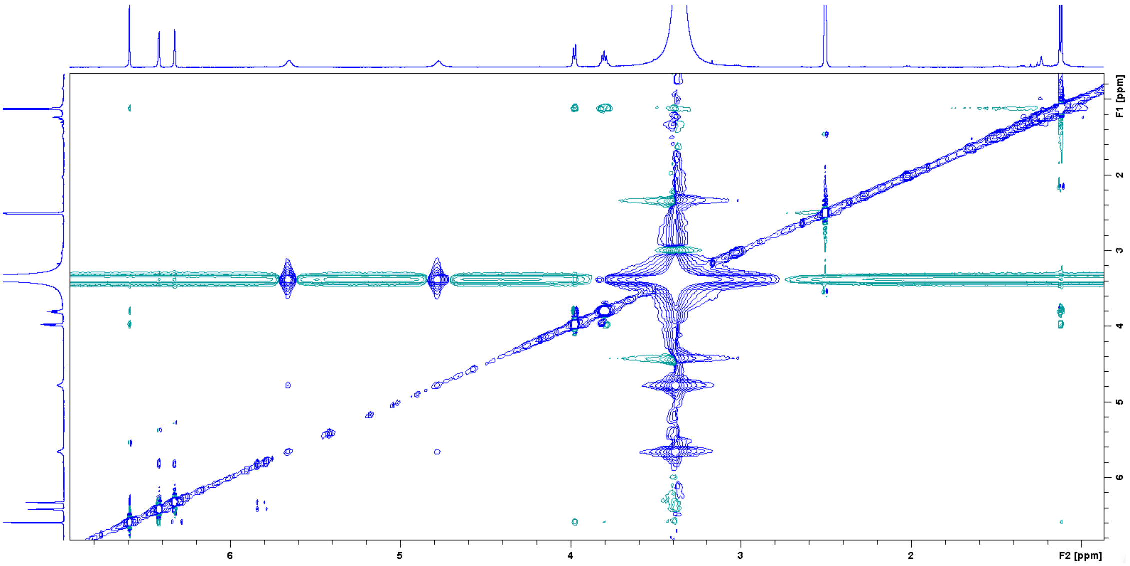

Figure S24. The HRESIMS spectrum of new compound **3** (*m/z* 275.0520 $[M+Na]^+$ (calcd for $C_{12}H_{12}NaO_6$, 275.0526))

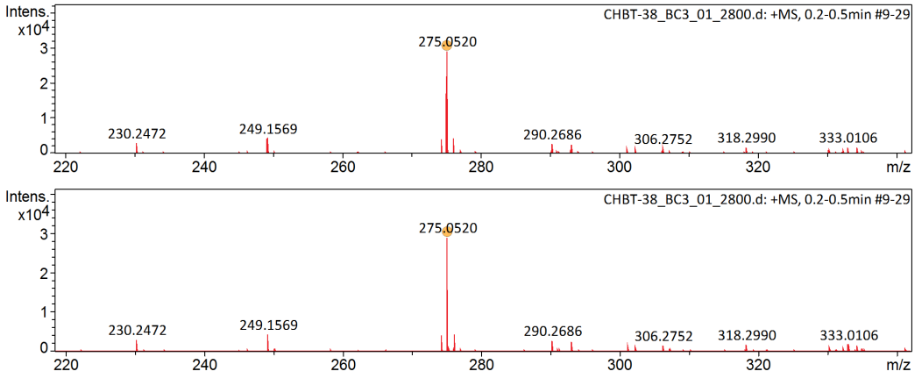

Meas. m/z	#	Ion Formula	m/z	err [ppm]	mSigma	# mSigma	Score	rdb	e⁻ Conf	N-Rule	Adduct
275.0520	1	C12H12NaO6	275.0526	2.3	9.3	1	100.00	7.0	even	ok	M+Na

Figure S25. ^{1}H NMR (500 MHz, CD_3OD) spectrum of new compound **4**

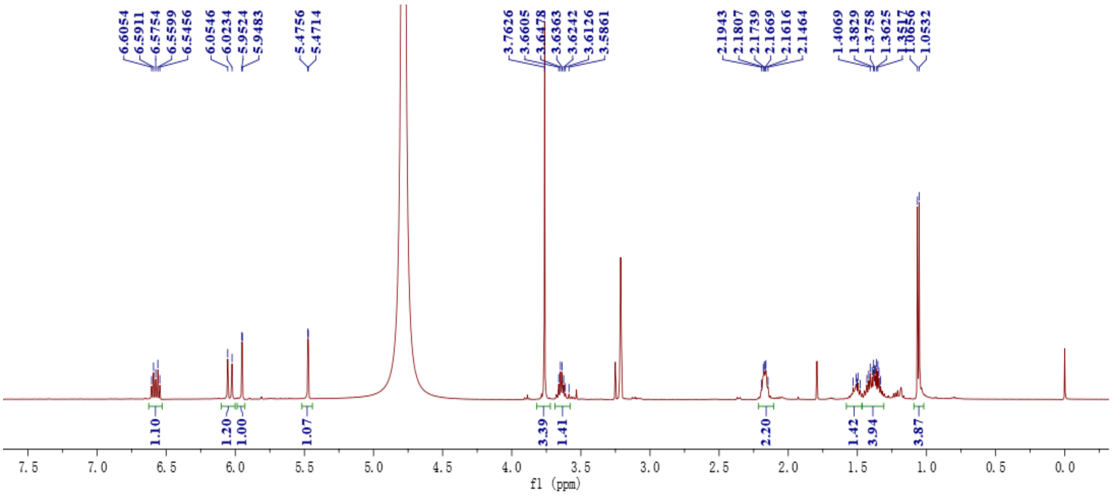

Figure S26. ^{13}C NMR (125 MHz, CD_3OD) spectrum of new compound **4**

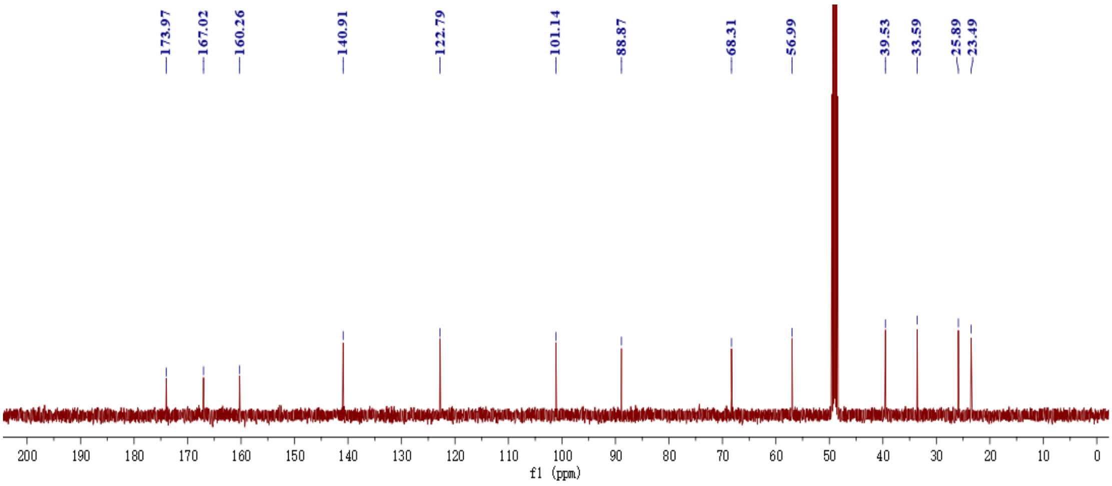

Figure S27. DEPT135 spectrum of new compound **4**

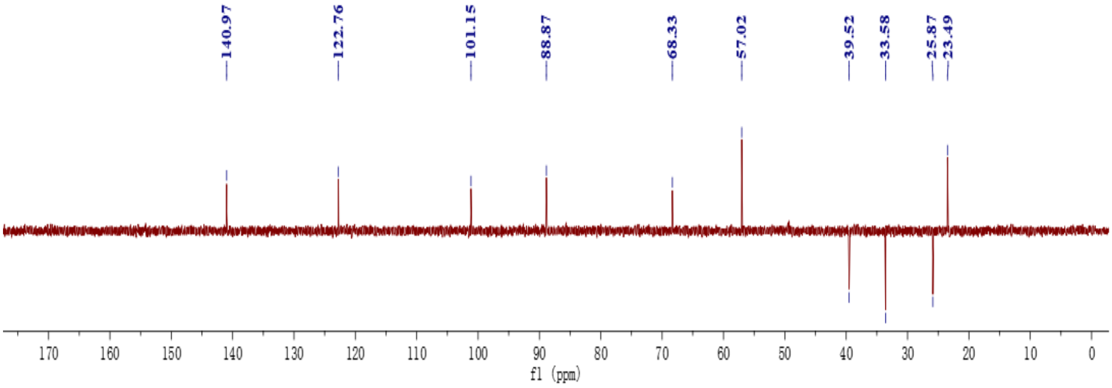

Figure S28. HSQC spectrum of new compound **4**

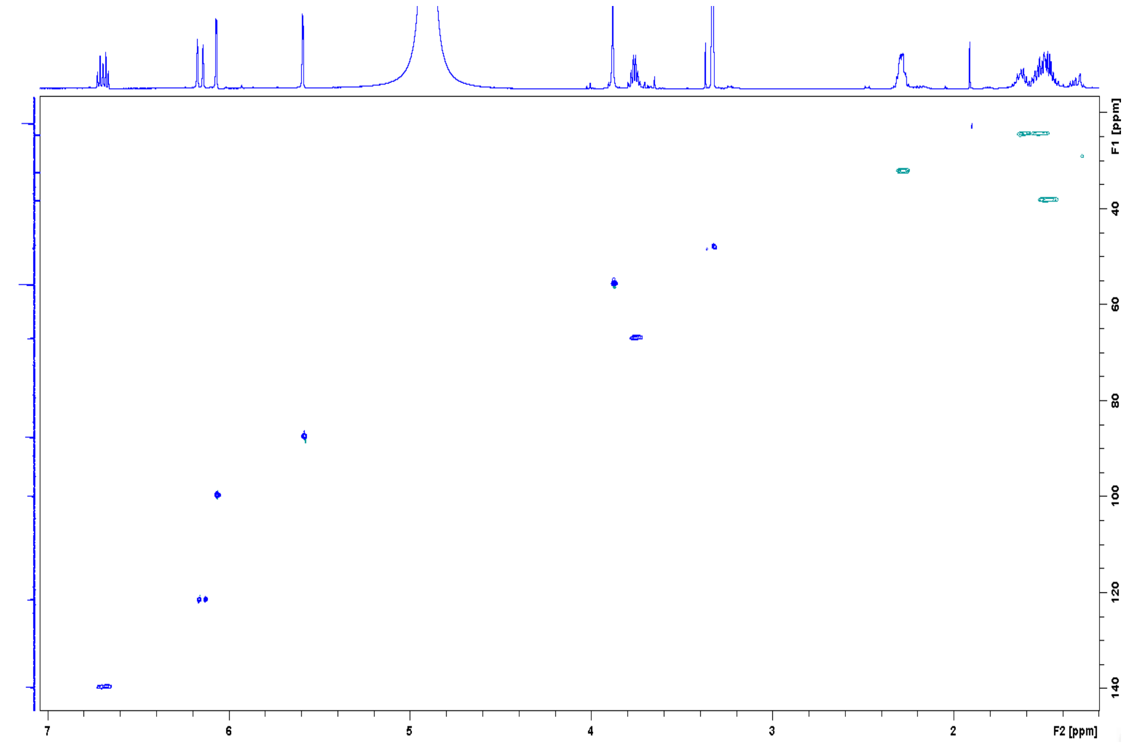

Figure S29. HMBC spectrum of new compound **4**

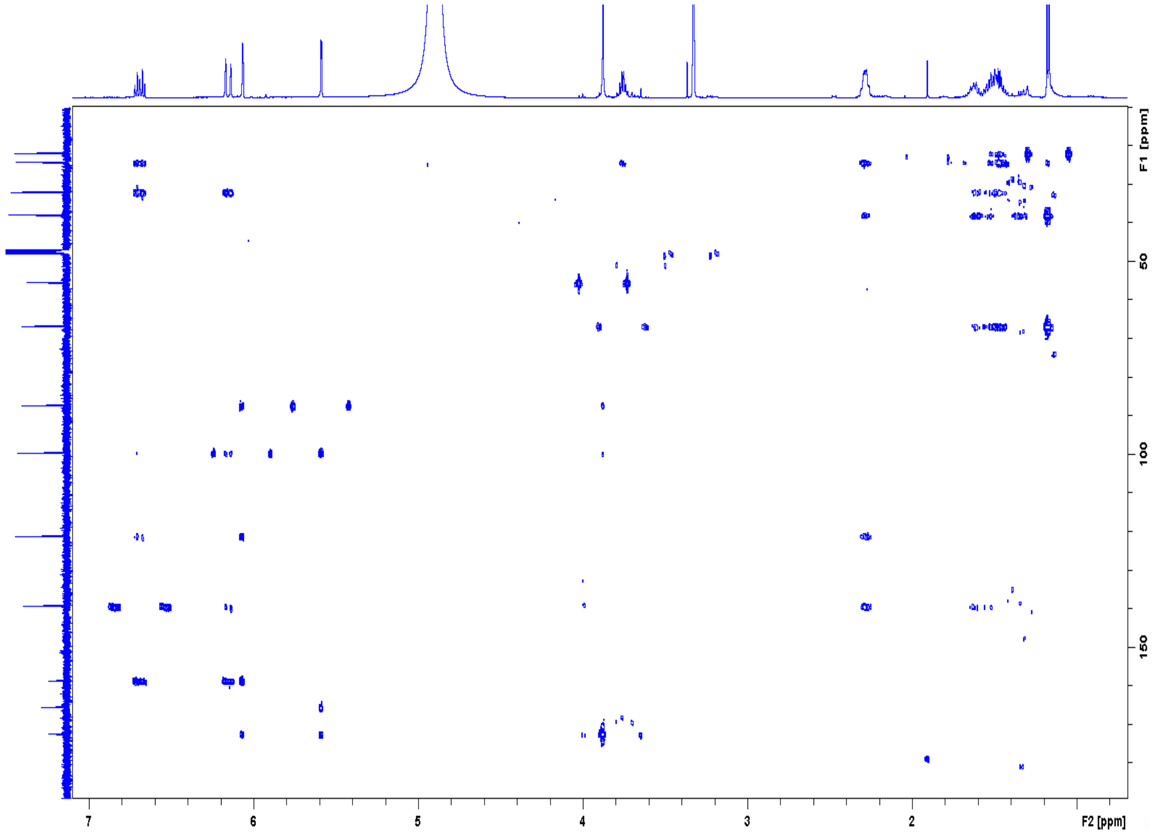

Figure S30. ^{1}H-^{1}H COSY spectrum of new compound **4**

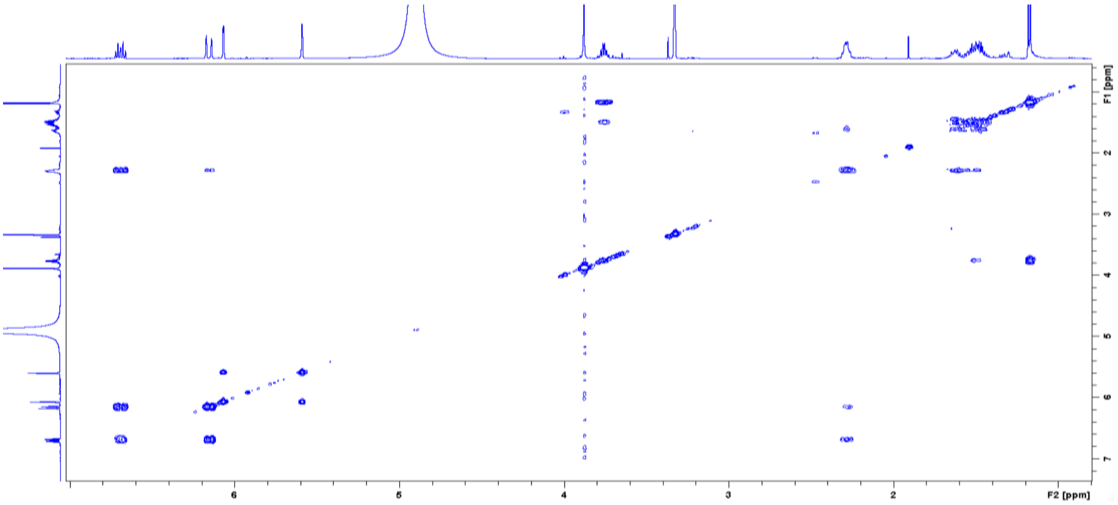

Figure S31. NOESY spectrum of new compound **4**

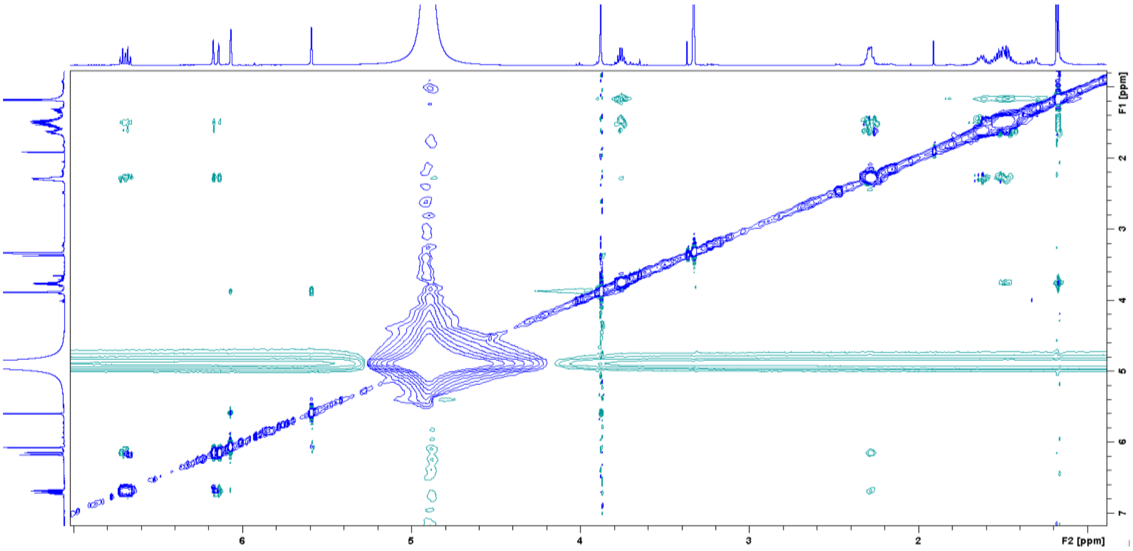

Figure S32. The HRESIMS spectrum of new compound **4** (*m/z* 261.1095 [M+Na]$^{+}$ (calcd for $C_{13}H_{18}NaO_4$, 261.1097))

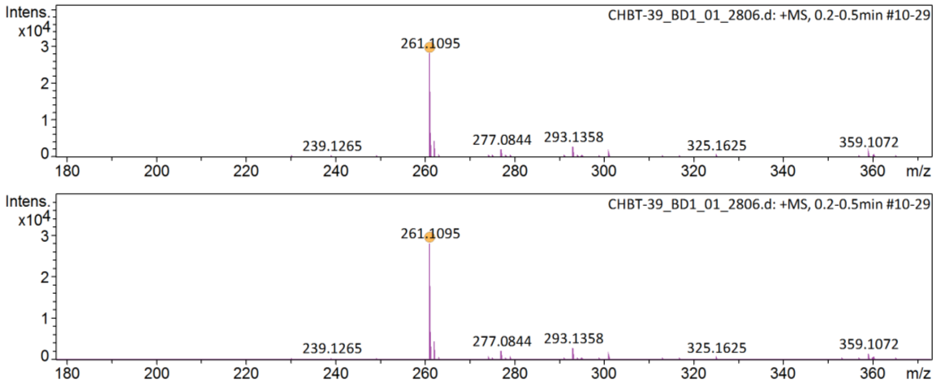

Meas. m/z	#	Ion Formula	m/z	err [ppm]	mSigma	# mSigma	Score	rdb	e⁻ Conf	N-Rule	Adduct
261.1095	1	C13H18NaO4	261.1097	1.0	7.3	1	100.00	5.0	even	ok	M+Na
